# Cystathionine-Gamma-Lyase Gene Deletion Protects Mice against Inflammation and Liver Sieve Injury following Polymicrobial Sepsis

**DOI:** 10.1371/journal.pone.0160521

**Published:** 2016-08-12

**Authors:** Ravinder Reddy Gaddam, Robin Fraser, Alireza Badiei, Stephen Chambers, Victoria C Cogger, David G Le Couteur, Isao Ishii, Madhav Bhatia

**Affiliations:** 1 Department of Pathology, University of Otago, Christchurch, New Zealand; 2 Centre for Education and Research on Ageing, Alzheimers and Ageing Institute, Biogerentology, ANZAC Research Institute, University of Sydney, Sydney and Concord Hospital, Sydney, Australia; 3 Department of Biochemistry, Graduate School of Pharmaceutical Sciences, Keio University, Tokyo, Japan; University of Szeged, HUNGARY

## Abstract

**Background:**

Hydrogen sulfide (H_2_S), produced by the activity of cystathionine-gamma-lyase (CSE), is a key mediator of inflammation in sepsis. The liver sinusoidal endothelial cells (LSECs) are important target and mediator of sepsis. The aim of this study was to investigate the role of CSE-derived H_2_S on inflammation and LSECs fenestrae in caecal-ligation and puncture (CLP)-induced sepsis using CSE KO mice.

**Methods:**

Sepsis was induced by CLP, and mice (C57BL/6J, male) were sacrificed after 8 hours. Liver, lung, and blood were collected and processed to measure CSE expression, H_2_S synthesis, MPO activity, NF-κB p65, ERK1/2, and cytokines/chemokines levels. Diameter, frequency, porosity and gap area of the liver sieve were calculated from scanning electron micrographs of the LSECs.

**Results:**

An increased CSE expression and H_2_S synthesizing activity in the liver and lung of wild-type mice following CLP-induced sepsis. This was associated with an increased liver and lung MPO activity, and increased liver and lung and plasma levels of the pro-inflammatory cytokines TNF-α, IL-6, and IL-1β, and the chemokines MCP-1 and MIP-2α. Conversely, CSE KO mice had less liver and lung injury and reduced inflammation following CLP-induced sepsis as evidenced by decreased levels of H_2_S synthesizing activity, MPO activity, and pro-inflammatory cytokines/chemokines production. Extracellular-regulated kinase (ERK1/2) and nuclear factor-κB p65 (NF-κB) became significantly activated after the CLP in WT mice but not in CSE KO mice. In addition, CLP-induced damage to the LSECs, as indicated by increased defenestration and gaps formation in the LSECs compared to WT sham control. CSE KO mice showed decreased defenestration and gaps formation following sepsis.

**Conclusions:**

Mice with CSE (an H_2_S synthesising enzyme) gene deletion are less susceptible to CLP-induced sepsis and associated inflammatory response through ERK1/2-NF-κB p65 pathway as evidenced by reduced inflammation, tissue damage, and LSECs defenestration and gaps formation.

## Introduction

Hydrogen sulfide (H_2_S) is a biologically active gaseous mediator that is biosynthesized from L-cysteine by the activity of cystathionine-γ-lyase (CSE), cystathionine-β-synthase (CBS) or 3-mercaptopyruvate sulfurtransferase (3-MST) [1]. CBS is predominantly expressed in the brain and kidney whereas CSE is more abundant in the liver, where it is the principal H_2_S-synthesizing enzyme [2]. H_2_S generated by CSE plays an important role in physiological and pathological processes [3]. It is a potent vasodilator/neurotransmitter and a pro-inflammatory mediator [4]. Alteration of the H_2_S/CSE signalling pathway is associated with various inflammatory diseases such as acute pancreatitis [4, 5], lipopolysaccharide (LPS)-induced endotoxemia [6, 7], severe burn injury [8], hind-paw edema [9], rheumatoid arthritis [10] as well as caecal-ligation and puncture (CLP)-induced sepsis [3, 11, 12]. These studies have shown that increased CSE expression/H_2_S synthesis, which promote inflammation by upregulating pro-inflammatory cytokines and chemokines [3–9]. The mechanism of CSE-derived H_2_S for inflammatory regulation involves activation of a transcription factor, NF-κB [12]. H_2_S activates ERK [13] by phosphorylation or NF-κB [12] by sulfhydration on cysteine-38 of the p65 subunit which upregulates the production of cytokines and chemokines [14].

Sepsis remains a critical clinical problem that is associated with significant morbidity and mortality. The causes of sepsis are multifactorial and is frequently associated with bacterial infections [15] often leading to a systemic inflammatory response and multiple organ failure [16]. During sepsis, the liver plays an important role in defensive responses by scavenging bacteria and producing inflammatory mediators [17]. The four main cell types which contribute to the hepatic defensive response are Kupffer cells (KCs), neutrophils and hepatocytes (by phagocytosis), and liver sinusoidal endothelial cells (LSECs) (by pinocytosis) [18, 19]. KCs are the primary defence against bacteria and endotoxemia. Excessive stimulation of KCs during endotoxemia causes them to release cytokines (TNF-α, IL-6, and IL-1β), which induce LSEC and hepatocyte injury [20]. LSECs also have the capacity to produce pro-inflammatory cytokines which are increased when LSECs are stimulated with LPS [21]. LSECs are unusual endothelial cells because they contain fenestrae of approximate diameters of 50–150 nm that regulate the process of filtration of substrates or particles between the sinusoidal lumen and subendothelial space of Disse. It has been reported that defenestration of LSECs is associated with endotoxemia-induced sepsis [22–24]. Severe lung injury is also associated with sepsis and with increased lung inflammation in CLP-induced sepsis [25].

Currently, there is limited information about the role of H_2_S/CSE on ERK1/2-NF-κB p65 signalling pathway and associated inflammation during sepsis. Numerous studies have shown the increased levels of CSE expression and H_2_S production during sepsis suggesting an important role of H_2_S in sepsis [3, 6, 7, 12, 13, 25, 26]. H_2_S promotes inflammation by increasing production of cytokines and chemokines during sepsis through the activation of ERK1/2-NF-κB p65 pathway [12, 13]. In addition, so far, inflammatory studies that inhibited endogenous H_2_S synthesis have employed the use of nonspecific CSE inhibitors, the most popular being DL-propargylglycine (PAG). Inhibition of CSE activity by prophylactic and therapeutic administration of PAG ameliorated inflammation by decreasing the synthesis of H_2_S and subsequent inflammatory cytokines and chemokines during sepsis [3, 6, 7, 11–13]. However, interpretation of results from CSE inhibitors such as PAG is limited as it has non-specific effects in addition to CSE inhibitory activity [8]. Apart from inhibiting CSE, PAG has also known to inhibit L-alanine transaminase and amino acid oxidases, and alter amino acid metabolism independent of CSE [27–29]. Furthermore, no study has, as yet, investigated the role of CSE/H_2_S signalling pathway on LSECs fenestrae during sepsis.

In the present study, we tested the hypothesis that H_2_S synthesized through CSE contributes to inflammation and changes LSEC ultrastructure in CLP-induced sepsis. To investigate this, CSE KO mice were used to study the effect of CSE deletion on liver and lung inflammation and injury, systemic inflammatory response and LSEC fenestrae in a mouse model of polymicrobial sepsis.

## Methods

### Induction of Sepsis

WT (C57BL/6J) mice were obtained from the Christchurch Animal Research Area (CARA) and CSE KO mice (on a C57BL/6J background) were generated by crossing CSE heterozygous mice as described previously [30]. WT and CSE KO mice (male, aged between 8–10 weeks; 25-30g) were randomly assigned to control and experimental groups. A total of 32 mice were used (WT sham, CSE KO sham, WT sepsis, and CSE KO sepsis (n = 8 each)). All experiments were approved by the Animal Ethics Committee of the University of Otago-Christchurch and were performed according to the established university guidelines.

CLP-induced sepsis is a severe animal disease model of sepsis with the evidence of maximum inflammatory changes at 8 hours in this model and mortality begins 24 hours after the CLP surgery as previously reported [3, 11]. We have performed CLP-induced sepsis procedure according to the previously described protocol with minor modifications [3, 11, 24, 31]. Mice were lightly anesthetized by inhaled isoflurane (using 2% isoflurane in 1L/min O_2_), buprenorphine (Temgesic, 0.2mg/kg SC) was administered 45 minutes prior to surgery and 3 hours after the surgery for analgesia. Sterile surgical techniques were used and a small midline incision was made through the skin and peritoneum of the abdomen to expose the caecum. The caecal appendage was ligated with silkam 5.0 thread at 8–10 mm from the tip of the caecum without occluding the bowel passage and then perforated in two evenly spaced locations at the distal end of the caecum with a 22 gauge needle. After this, a small amount of stool was squeezed out through each holes. Finally, the bowel was repositioned, and the abdomen was sutured with sterile permilene 5.0 thread. Sham mice underwent the same procedure without CLP. 8 hours after surgery the mice were euthanized by an intraperitoneal (IP) injection of sodium pentobarbital (150 mg/kg). Blood samples were withdrawn from the right ventricle using heparinized syringes. Plasma was collected following centrifugation (1,000g for 5 minutes, at 4 ^o^C). Plasma was then stored at -80°C for subsequent measurement of cytokines and chemokines by Enzyme Linked Immuno Sorbent Assay (ELISA). Random pieces of the liver and lungs were fixed in 10% wt/vol neutral phosphate-buffered formalin, dehydrated through a graded ethanol series, and embedded in paraffin wax for histology. The remaining liver and lung samples were stored at -80 ^o^C for measurement of cytokines and chemokines by ELISA, tissue myeloperoxidase (MPO) and H_2_S synthesizing activity by spectrophotometric assay, CSE and ERK1/2 protein expression by western blotting and NF-κB p65 activity by NF-κB p65 DNA binding activity assay.

### Post-operative Animal Care

After CLP or sham operation, mice were returned to the temperature (37 ^o^C) regulated (with heating pads) original mice cages at the surgical area. Mice were observed 15 minutes after surgery and then every 1 hour until sacrifice. 1 ml of warm sterile saline was injected SC to help avoid dehydration directly after surgery and 3 hours after surgery. Analgesic, buprenorphine (Temgesic, 0.2mg/kg SC), was administered 45 minutes before and 3 hours after the surgery to control pain. All the conditions of the animal well-being such as physical parameters (body weight change and water consumed), general clinical signs (dehydration, activity levels, hunched posture and rough coat) and behavioural signs of pain and stress (back arch, belly press, writhe, stagger and fall) were recorded (with scoring normal mice as 0; score 1, 2, 3 for increase in severity) for every 1 hour. Condition of surgical sites were also monitored for wound or sutures leaking and bleeding for every 1 hour.

Mice with CLP operation showed more body weight loss (but within the 10% weight loss of human end point) compared to sham mice. We also monitored for general clinical signs of well-being and behavioural signs of pain and stress which were severe in mice with CLP operation (with the severity score of 1–2) than sham group (with the severity score of 0–1). However, we have not observed any bleeding, and wound or suture leaking at the surgical sites and mortality in both CLP and sham operated mice prior to the experimental endpoint.

### Western Blotting

Liver and lung tissue lysates were prepared by homogenization in ice-cold RIPA buffer supplemented with a protease inhibitor cocktail (Halt; Thermo Scientific Pierce Protein Biology, Rockford, IL). The resulting homogenates were then rocked at 4°C for 30 minutes before centrifuging at 10,000g for 10 minutes at 4°C. The clear lysates were then stored at -80°C until further use. A sample (15 μg) of protein from each sample was separated on a 10% SDS-PAGE gel under reducing conditions. Gels were transferred onto a 0.45 μm nitrocellulose membrane (Protran by Whatman) via a wet transfer using Towbins buffer supplemented with 10% methanol for 1 hour. Membranes were then blocked for 1 hour followed by overnight incubation with the primary antibody (1:1,000 for CSE, 1:2,000 for anti-ERK1/2 and anti-p-ERK1/2) at 4°C, followed by 2 hour incubation with the secondary antibody (1:10,000 for both CSE and ERK1/2) at room temperature, and detection with a chemiluminescent substrate (Supersignal West Pico, Thermo Scientific Pierce Protein Biology). Detection and quantification was performed on a chemi-doc system (Uvitec, Cambridge, UK). Blocking buffer consisted of Tris-buffered saline with 0.1% wt/vol Tween-20 (TBST) and 5% wt/vol non-fat dry milk. All antibodies were prepared in a blocking buffer and washings were done with TBST only. Mouse anti-human CSE was purchased from Abnova (Taipei City, Taiwan), and anti-ERK1/2 and anti-p-ERK1/2 were purchased from cell signaling (Danvers, MA). Rabbit anti-mouse glyceraldehyde-3-phosphate dehydrogenase (GAPDH), goat anti-rabbit horseradish peroxidase (HRP)-conjugated antibody and goat anti-mouse HRP-conjugated antibody were purchased from Santa Cruz Biotechnology (Santa Cruz, CA).

### H_2_S-Synthesizing Activity Assay

H_2_S-synthesizing activity in the liver and lung homogenates was measured with a modified protocol based on methods described previously [32]. Briefly, the liver and lung tissues were homogenized in 20 mM ice-cold sodium phosphate buffer (pH 7.4) with protease inhibitors. The reaction mixture contained tissue homogenate (230 μl) in 20 mM sodium phosphate buffer (pH 7.4), L-cysteine (10 μl, 250 mM), and pyridoxyal 5’-phosphate (10 μl, 18 mM). The reaction was performed in tightly parafilm-sealed microfuge tubes and initiated by transferring the tubes from ice to a shaking water bath at 37 ^o^C. After incubation for 30 minutes, 1% wt/vol zinc acetate (125 μl) was injected in to trap evolved H_2_S. Subsequently, a mixture of N, N-dimethyl-p-phenylenediamine sulfate (20 mM) in 7.2 M HCl and FeCl_3_ (30 mM) in 1.2 M HCl (133 μl, in 1:1 ratio) was added. Samples were left to incubate at room temperature in the dark for 20 minutes. Followed this 10% vol/vol trichloroacetic acid (25 μl) which was added to denature the protein and stop the reaction. After centrifugation, the absorbance of the supernatant was measured with a 96-well microplate spectrophotometer at 670 nm. The H_2_S concentration was calculated against a calibration curve of Na_2_S. Results were then corrected for the protein content of the tissue sample determined by the Bradford assay and are expressed as nmole H_2_S formed/mg protein.

### Scanning Electron Microscopy (SEM)

Eight hours after sham or CLP operation, mice were anaesthetized by single i.p. injection of sodium pentobarbital (80 mg/kg). The abdomen was opened and the liver was perfused by cannulating the portal vein with a 22 g needle attached to a perfusion apparatus as described previously [33]. Briefly, livers were perfusion fixed with 2.5% glutaraldehyde in 0.1 M sodium cacodylate buffer. Perfusion fixed liver specimens were cut into small pieces followed by incubation with 0.1 M Cac buffer/2% sucrose, 1% tannic acid/2% sucrose, 1% osmium/2% sucrose pH 7.4, and a series of ethanol (50%, 70%, 90%, 95%, and 100%) and finally with hexamethyldisilazane (HMDS). The liver specimen blocks were mounted onto the SEM slug mounts and coated with platinum using the sputter coater. The coated blocks were examined with JSM6380 SEM (JEOL, Japan) at 15 kV acceleration voltages. Fenestration number and diameter were measured, together with total area assessed, using Image J (NIH). These data were used to calculate the porosity (the percentage of the LSEC surface area covered with fenestrations [33].

### Myeloperoxidase Activity (MPO)

Leukocyte sequestration in the liver and lung was quantified by measuring tissue MPO activity. Tissue samples were thawed, homogenized in 20 mM phosphate buffer (pH 7.4), centrifuged (10,000g, 10 minutes, 4°C) and the resulting pellet was re-suspended in 50 mM phosphate buffer (pH 6.0) containing 0.5% w/v hexadecyltrimethylammonium bromide (Sigma-Aldrich). Three cycles of freezing and thawing carried on the suspension followed by sonication for 40 seconds. The samples were then centrifuged (10,000g, 5 minutes, 4°C) and the supernatants were used for the MPO assay. The reaction mixture consisted of the supernatant (50 μl), 1.6 mM tetramethylbenzidine, 80 mM sodium phosphate buffer (pH 5.4) and 0.3 mM hydrogen peroxide (reagent volume: 100 μl). This mixture was incubated at 37°C for 110 seconds. The reaction was terminated with 50 μl of 2 M H_2_SO_4_. The absorbance was measured at 450 nm and corrected for the protein content of the tissue sample using results from the DC protein assay. The results were expressed as fold increase over control.

### Histological Analysis

Tissue samples were fixed with 10% buffered formaldehyde and embedded in paraffin. Liver and lung sections (5 μm) were stained with haematoxylin and eosin (H&E) using standard protocols. Liver sections of histology slides were assessed for capsular inflammation and lobular necrotic damage using modified Knodell Histology Activity Index (HAI) of blinded scoring system of liver injury [34, 35]. Lung sections were assessed for leukocyte infiltration and alveolar wall thickening using blinded lung injury scoring system suggested by American Thoracic Society guidelines [36] using a Leica microscope at 20x magnification.

### NF-*κ*B Activity Assay

The binding of NF-*κ*B p65 to DNA was measured in nuclear extract of liver and lung tissues using an ELISA based TransAmTMNF-*κ*B p65 transcription factor assay kit (Active Motif) as per the manufacturer’s instructions (Wangarra, Australia). Briefly, nuclear extracts (20 *μ*g) were incubated in a 96-well plate with complete lysis buffer for 1 hour followed by incubation with a specific primary antibody against NF-*κ*B p65 for 1 hour. Subsequently, a horse radish peroxidase-(HRP)-conjugated secondary antibody was used for detection. The enzymatic product was measured at 450 nm using a microplate reader. The specificity of the assay was determined by wild-type or mutated comparative control wells. Results were expressed as fold increase over control groups.

### Enzyme-linked Immunosorbent Assay

An Enzyme-Linked Immunosorbent Assay (ELISA) was performed to determine the protein levels of the main liver, lung and plasma pro-inflammatory cytokines and chemokines (TNF-α, IL-6, IL-1β, MCP-1 and MIP-2α) according to the manufacturer’s protocol supplied by R&D Systems (Minneapolis, MN). To prepare the liver and lung homogenates, 50 mg tissue was homogenized in 1 ml 20 mM sodium phosphate buffer, pH 7.4 on ice. Homogenates were centrifuged at 10,000g for 10 minutes at 4°C. The supernatant was used to measure the levels of TNF-α, IL-6, IL-1β, MCP-1 and MIP-2α. Measurements were corrected for the protein content of the tissue sample using results from the DC protein assay and expressed as nanogram per milligram of protein or nanogram per mL of plasma. The lower limits for detection of these cytokine and chemokines levels as follows: TNF-α, 31.3 pg/mL; IL-6, 15.6 pg/mL; IL-1β, 15.6 pg/mL; MCP-1, 3.91 pg/mL; and MIP-2α, 15.6 pg/mL.

### Statistical Analysis

Data are expressed as mean ± standard deviation. All the experimental data were analysed for Gaussian or Normal distribution using Shapiro-Wilk test. One-way ANOVA with post hoc Tukey’s test was performed with the Gaussian distribution data and non-parametric Kruskal-Wallis test was performed with the data which were not followed Gaussian distribution to compare multiple groups using GraphPad version 6.3. A P<0.05 was considered statistically significant.

## Results

### CSE Protein Expression and H_2_S-Synthesizing Activity

**Fig 1:**
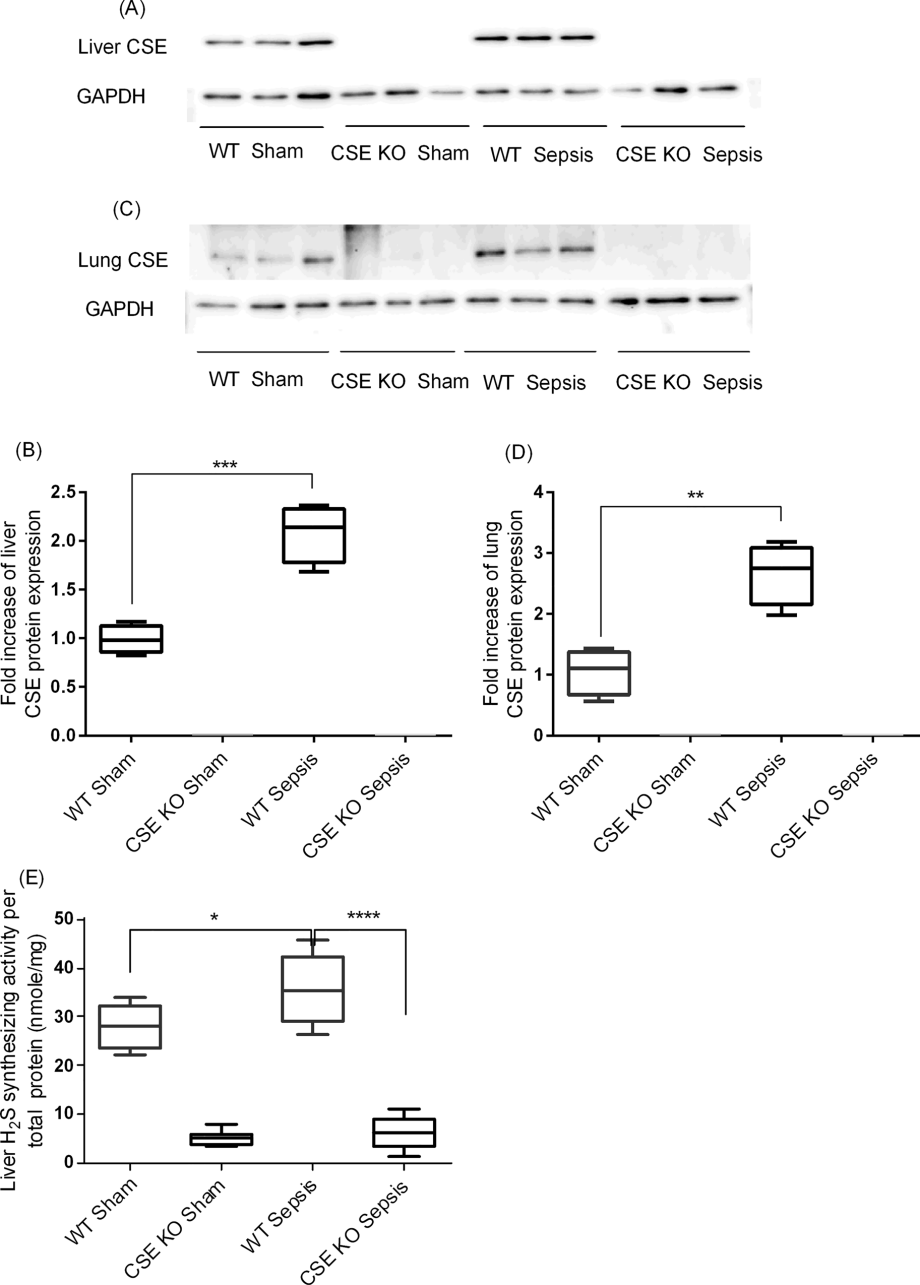
CSE Protein Expression and H_2_S-Synthesizing Activity Following CLP Induced Sepsis. (A-B) Liver CSE protein expression and (C-D) lung CSE protein expression. Liver and lung CSE protein expression was increased following CLP induced sepsis compared to sham control (liver: P<0.001 vs. sham control; lung P<0.01 vs. sham control) and no CSE protein expression was detected in CSE KO mice. Results were normalized with GAPDH and expressed as the relative fold increase of CSE protein expression compared with sham control. For western blot results, each lane represents a separate animal. The blots shown were representative of all animals in each group with similar results. (E) Liver H_2_S synthesizing activity. H_2_S synthesizing activity was increased following increased CSE protein expression in WT CLP induced sepsis mice compared to sham controls and CSE KO mice had significantly lower H_2_S synthesizing activity compared to WT sepsis mice. Data represent the mean±standard deviation (n = 8). Data were analysed for Gaussian or Normal distribution using Shapiro-Wilk test. One-way ANOVA with post hoc Tukey’s test was performed to compare multiple groups. Statistical significance was assigned as *P<0.05; **P<0.01; ***P<0.001; and ****P<0.0001.

CLP-induced sepsis was associated with significantly increased levels of CSE protein expression in both liver and lung of WT mice compared to sham-operated control mice (Fig 1A–1D). As expected, CSE protein expression was not detectable in both liver and lung of CSE KO mice (Fig 1A–1D). In accordance, the sepsis was associated with increased H_2_S-synthesizing activity in the liver of WT mice (Fig 1E). There was much lower levels of liver H_2_S-synthesizing activity in CSE KO mice and the sepsis did not cause significant increases in it in CSE KO mice (Fig 1E). We failed to detect any measurable levels of H_2_S synthesizing activity in the lung tissue homogenates.

### Effect of CSE Deletion on Leukocyte Infiltration and Sepsis-Associated Organ Injury

**Fig 2:**
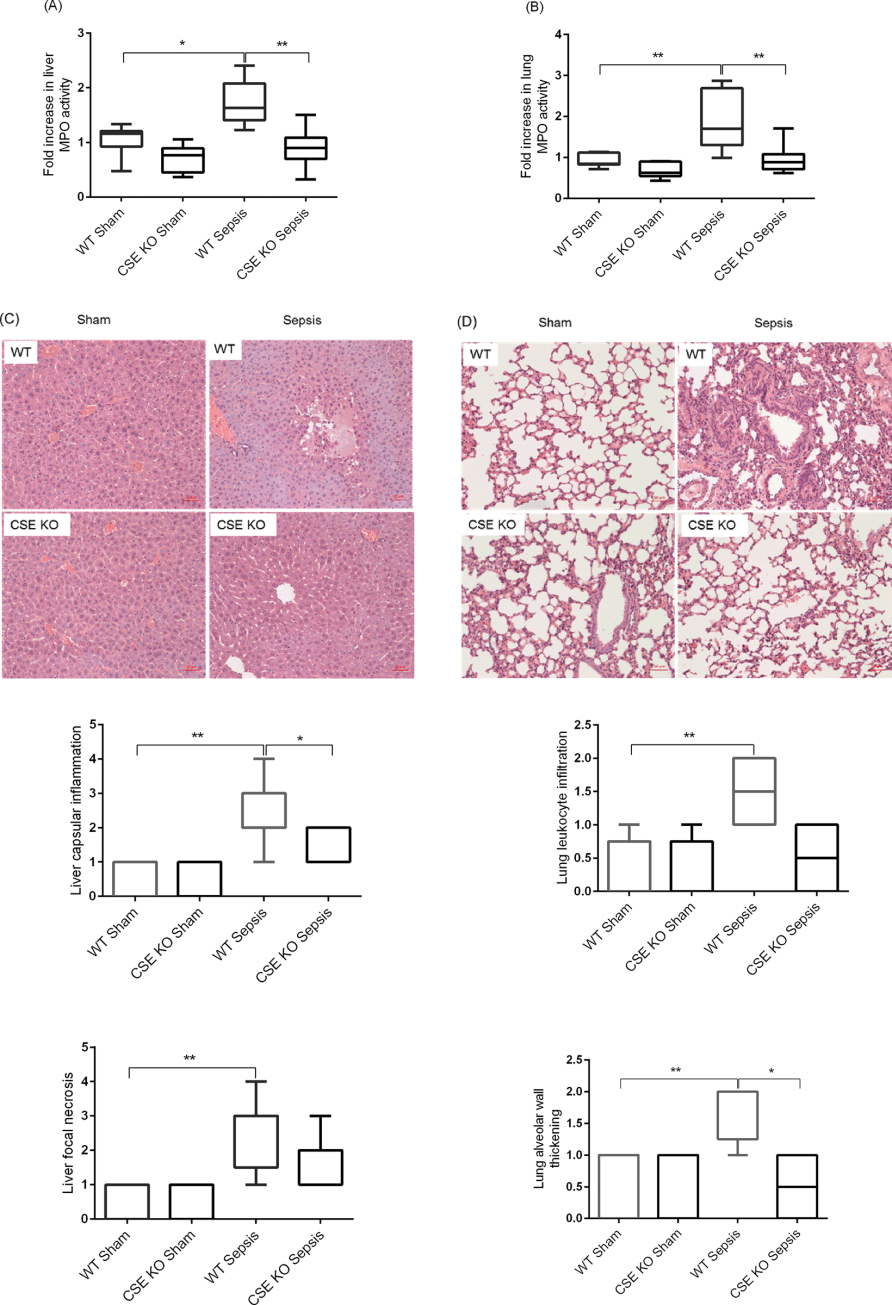
Effect of CSE Gene Deletion on Liver and Lung MPO Activity and Organ Injury in Mice Following CLP Induced Sepsis. (A) Liver MPO activity and (B) Lung MPO activity. Following CLP induced sepsis, liver and lung MPO activity levels were increased in WT sepsis mice compared to sham operation controls. CSE gene deletion decreased significantly liver and lung MPO activity following CLP induced sepsis compared to WT sepsis mice. Results were expressed as the relative fold increase of MPO activity compared with sham operation controls. (C) Representative images of the liver H&E sections revealed extensive capsular inflammation and lobular necrosis in CLP induced sepsis in the WT mice compared to sham control. The CSE KO mice showed a lower capsular inflammation and lobular necrosis. (D) Representative images of the lung H&E sections. Histological examination of the lung sections reveal marked leukocyte infiltration and alveolar thickening following CLP induced sepsis in the WT mice compared to sham operation controls. This effect was substantially reduced in the CLP induced CSE KO mice. Scale bar is 50 μm. Data represent the mean±standard deviation (n = 8). Data were analysed for Gaussian or Normal distribution using Shapiro-Wilk test. Liver and lung MPO activity data were analysed using One-way ANOVA with post hoc Tukey’s test whereas liver and lung histology scores were analysed with non-parametric Kruskal-Wallis test to compare multiple groups. Statistical significance was assigned as *P<0.05; and **P<0.01.

Leukocyte infiltration was assessed by measuring liver and lung MPO activity because the rise in MPO activity reflects leukocyte infiltration into these organs. CLP-induced sepsis was associated with elevated MPO activity in both liver and lung of WT mice compared to sham-operated control mice (Fig 2A and 2B). The sepsis KO mice showed significantly less MPO activity in the liver and lungs compared with the WT sepsis mice (Fig 2A and 2B). Histological examination of WT sepsis liver identified apparent tissue damage (capsular inflammation and lobular necrosis) compared to WT sham control as well as CSE KO sepsis (and CSE KO sham control) (Fig 2C). H&E staining of lung sections were also revealed higher leukocyte infiltration and thickening of alveolar wall in WT sepsis mice than CSE KO sepsis mice, although normal lung histoarchitecture was observed in sham-operated WT and CSE KO mice (Fig 2D).

### Effect of CSE Deletion on Liver Sieve Injury Following Sepsis

**Fig 3:**
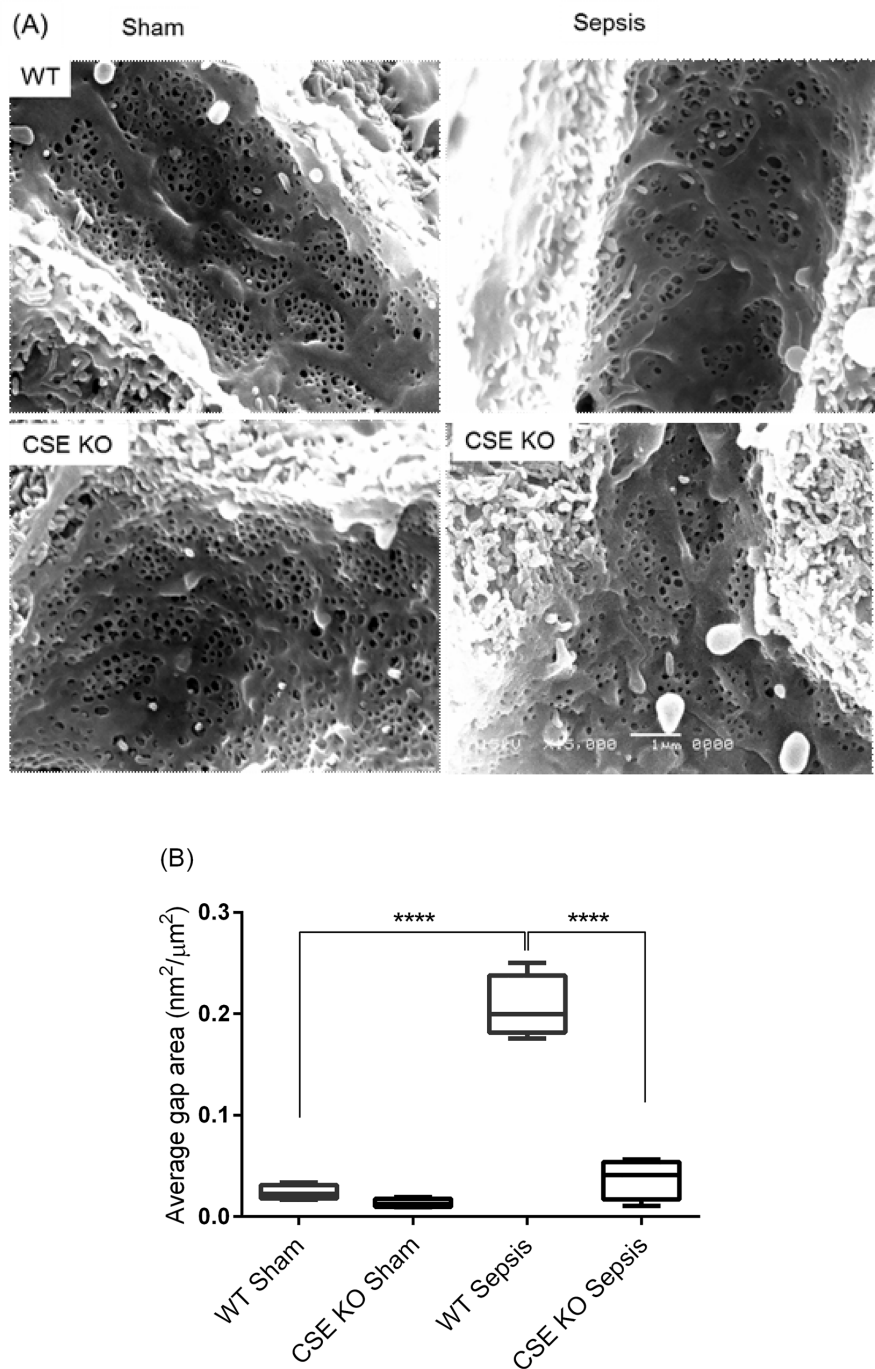
Effect of CLP Induced Sepsis and CSE Gene Deletion on Liver Sieve Injury in Mice. (A) Representative images of the LSECs micrographs of scanning electron microscopy of perfusion fixed liver sections after 8 hours CLP or sham operation (voltage and magnification: 15kV x15000; n = 8) and (B) Average graph area in the LSECs. Scanning electron microscopy studies revealed that LSECs injury was increased following 8 hours CLP surgery in WT mice as evidenced by increased gaps formation in LSECs. CSE KO mice had significantly fewer gaps compared to WT sepsis mice. Data represent the mean±standard deviation (n = 8). Data were analysed for Gaussian or Normal distribution using Shapiro-Wilk test. One-way ANOVA with post hoc Tukey’s test was performed to compare multiple groups. Statistical significance was assigned as ****P<0.0001.

Scanning electron micrographs show that an increased LSECs injury in WT CLP mice compared to WT sham group, as evidenced by decreased diameter, frequency, and porosity and increased gaps formation in the LSECs (Fig 3A and 3B). CSE KO CLP mice had significantly fewer gaps and increased diameter, frequency and porosity than WT CLP mice. There was no significant difference between CLP and sham groups in CSE KO mice (Table 1).

**Table 1:** Effect of CLP Induced Sepsis and CSE Gene Deletion on the Alteration of the LSECs Fenestration.

*Group*	*Diameter of fenestrae (nm)*	*Number of fenestrae/μm2*	*Porosity (%)*
***WT Sham***	*137.63± 6.46*	*8.42± 0.50*	*12.51± 0.59*
***CSE KO Sham***	*118.51± 3.07*	*8.44± 1.00*	*10.49± 0.66*
***WT Sepsis***	123.45± 3.13*	6.56± 0.28*	8.40± .67**
***CSE KO Sepsis***	*125.91± 5.42*	*8.92± 0.68^#^*	*11.27± 1.14^##^*

CLP induced sepsis mice showed decreased dimeter, frequency, and porosity of LSECs fenestrae 8 hours after CLP compared to sham operation. Mice with CSE gene deletion have increased diameter, frequency and porosity of LSECs fenestrae compared WT mice following CLP sepsis. Data represent the mean±standard deviation (n = 8). Data were analysed for Gaussian or Normal distribution using Shapiro-Wilk test. One-way ANOVA with post hoc Tukey’s test was performed to compare multiple groups. Statistical significance was assigned as

* P<0.05

** P<0.01 vs WT sham

^#^ P<0.05

^##^ P<0.01 vs WT Sepsis.

### Effect of CSE Deletion on Liver and Lung ERK1/2-NF-κB p65 Activation Following Sepsis

**Fig 4:**
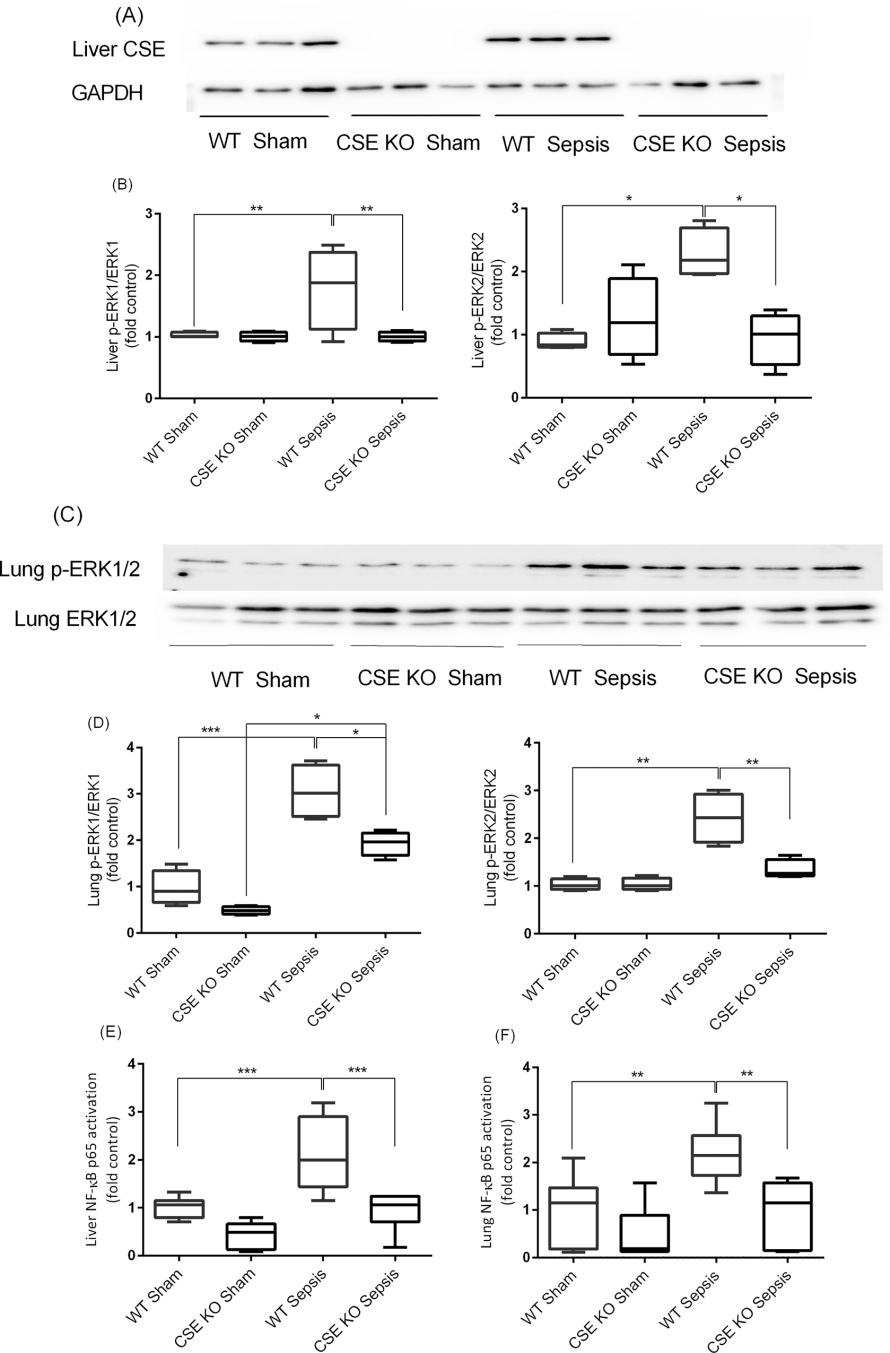
Effect of CSE Gene Deletion on the Phosphorylation of ERK1/2 and NF-kB p65 Activation in the Liver and Lung Following CLP Induced Sepsis. (A-B) Liver p-ERK1/2 expression. Phosphorylation of ERK1/2 (p-ERK1: P<0.01; p-ERK2: P<0.05) was increased following CLP induced sepsis in WT mice compared to sham operation controls. In CSE KO mice ERK1/2 phosphorylation (p-ERK1: P<0.01; p-ERK2: P<0.05) was reduced significantly following CLP induced sepsis compared to WT sepsis mice. (C-D) Lung p-ERK1/2 expression. Phosphorylation of ERK1/2 was increased (p-ERK1: P<0.001; p-ERK2: P<0.01) following CLP induced sepsis in WT mice compared to sham control. In CSE KO mice ERK1/2 phosphorylation was reduced significantly (p-ERK1: P<0.05; p-ERK2: P<0.01) following CLP induced sepsis compared to WT sepsis mice. Results were normalized with GAPDH and expressed as the relative fold increase of pERK1/2 expression compared with sham control. For western blot results, each lane represents a separate animal. The blots shown were representative of all animals in each group with similar results. (E-F) Liver and lung NF-κB p65 activation. Activation of NF-κB p65 (P<0.001) was increased following CLP induced sepsis in WT mice compared to sham control. In CSE KO mice NF-κB p65 activation (P<0.001) was decreased significantly following CLP induced sepsis compared to WT sepsis mice. Results were expressed as fold increase over control. Data represent the mean±standard deviation (n = 8). Data were analysed for Gaussian or Normal distribution using Shapiro-Wilk test. One-way ANOVA with post hoc Tukey’s test was performed to compare multiple groups. Statistical significance was assigned as *P<0.05; **P<0.01: and ***P<0.001.

Both WT and CSE KO sham mice had low basal activation of ERK1/2 in liver and lungs, as evidenced by decreased phosphorylation of ERK1/2. Following CLP induced sepsis, the phosphorylation of ERK1/2 significantly increased in the WT mice compared to sham control. The CLP-induced sepsis in CSE KO mice, however, had significantly less phosphorylation compared with the corresponding WT mice (Fig 4A–4D). This indicates a lower level of the liver and lung phosphorylation of ERK1/2 is due to CSE deletion in response to sepsis. Activation of the liver and lung NF-κB p65 was increased following CLP induced sepsis in WT mice compared to sham control. CSE gene deletion decreased the activation of NF-κB p65 in the liver and lungs following sepsis as compared to WT sepsis mice (Fig 4E–4F).

### Effect of CSE Deletion on Liver Pro-inflammatory Cytokines and Chemokines Production Following Sepsis

**Fig 5:**
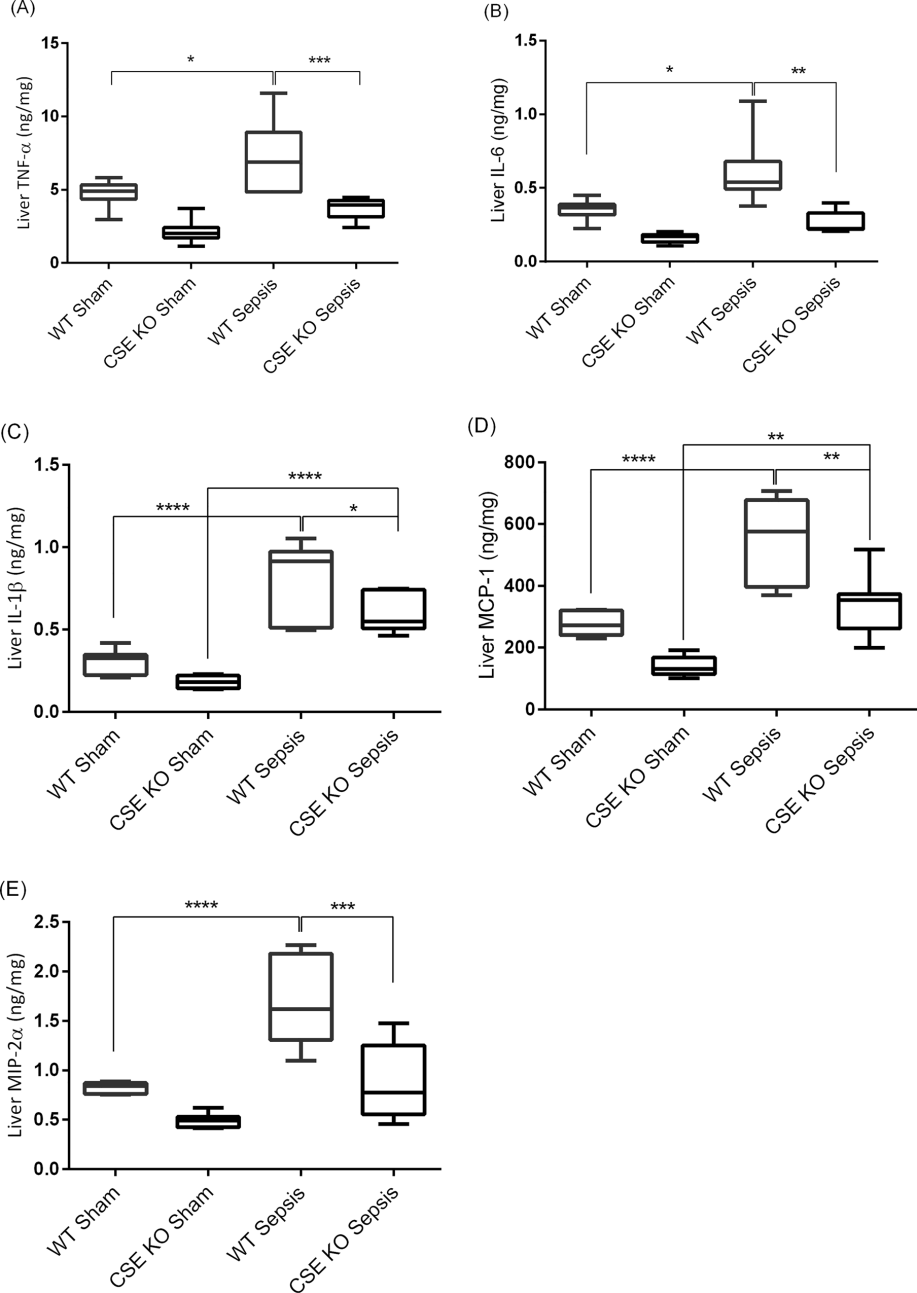
Effect of CSE Gene Deletion on Liver Pro-inflammatory Cytokine and Chemokine Levels Following CLP Induced Sepsis. (A) Liver TNF-α, (B) Liver IL-6, (C) Liver IL-1β, (D) Liver MCP-1 and (E) Liver MIP-2α. CLP induced sepsis significantly increased the liver cytokines TNF-α (P<0.05), IL-6 (P<0.05) and IL-1β (P<0.0001) and the chemokines MCP-1 (P<0.0001) and MIP-2α (P<0.0001) in WT mice compared to sham operation controls. Knockdown of the CSE gene protects mice against liver injury by reducing pro-inflammatory TNF-α (P<0.01), IL-6 (P<0.01), IL-1β (P<0.05), MCP-1 (P<0.01) and MIP-2α (P<0.001) following CLP induced sepsis compared to WT sepsis mice. Results were expressed in ng/mg of protein. Data represent the mean±standard deviation (n = 8). Data were analysed for Gaussian or Normal distribution using Shapiro-Wilk test. One-way ANOVA with post hoc Tukey’s test was performed to compare multiple groups. Statistical significance was assigned as *P<0.05; **P<0.01: ***P<0.001; and ****P<0.0001.

WT CLP mice showed significantly higher levels of liver pro-inflammatory cytokines and chemokines compared with WT sham mice (Fig 5A–5E). CSE KO CLP mice higher (or equivalent for TNF-α, IL-6, and MIP-2α) levels of those cytokines/chemokines in liver compared with CSE KO sham mice, but the levels were much lower than those of WT CLP mice (Fig 5A–5E).

### Effect of CSE Deletion on Lung Pro-inflammatory Mediators Following CLP-induced Sepsis

**Fig 6:**
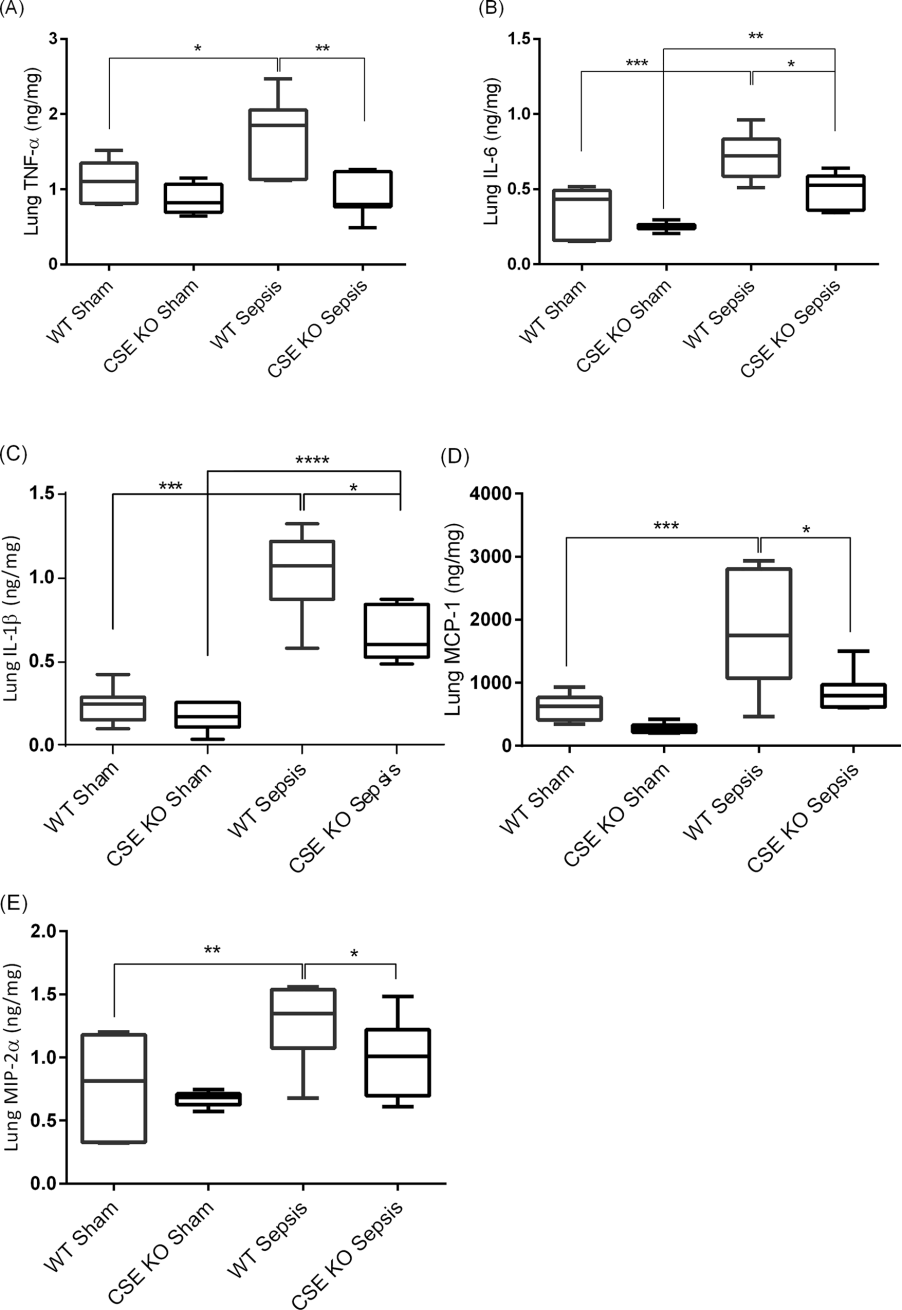
Effect of CSE Gene Deletion on Lung Pro-inflammatory Cytokines and Chemokines. (A) Lung TNF-α, (B) Lung IL-6, (C) Lung IL-1β, (D) Lung MCP-1 and (E) Lung MIP-2α. CLP induced sepsis significantly increased the lung cytokines TNF-α (P<0.05), IL-6 (P<0.001) and IL-1β (P<0.001) and the chemokines MCP-1 (P<0.001) and MIP-2α (P<0.01) in WT mice compared to sham operation controls. Knockdown of the CSE gene protects mice against lung injury by reducing pro-inflammatory TNF-α (P<0.01), IL-6 (P<0.05), IL-1β (P<0.05), MCP-1 (P<0.05) and MIP-2α (P<0.05) following CLP induced sepsis compared to WT sepsis mice. Results were expressed in ng/mg of protein. Data represent the mean±standard deviation (n = 8). Data were analysed for Gaussian or Normal distribution using Shapiro-Wilk test. One-way ANOVA with post hoc Tukey’s test was performed to compare multiple groups. Statistical significance was assigned as *P<0.05; **P<0.01: and ***P<0.001.

WT CLP mice showed significantly higher levels of lung pro-inflammatory cytokines and chemokines compared with WT sham mice (Fig 6A–6E). CSE KO CLP mice displayed higher (or equivalent for TNF-α, MCP-1, and MIP-2α) levels of those cytokines/chemokines in lungs compared with CSE KO sham mice, but the levels were much lower than those of WT CLP mice (Fig 6A–6E).

### Effect of CSE Deletion on Plasma Pro-inflammatory Mediators Following CLP-induced Sepsis

**Fig 7:**
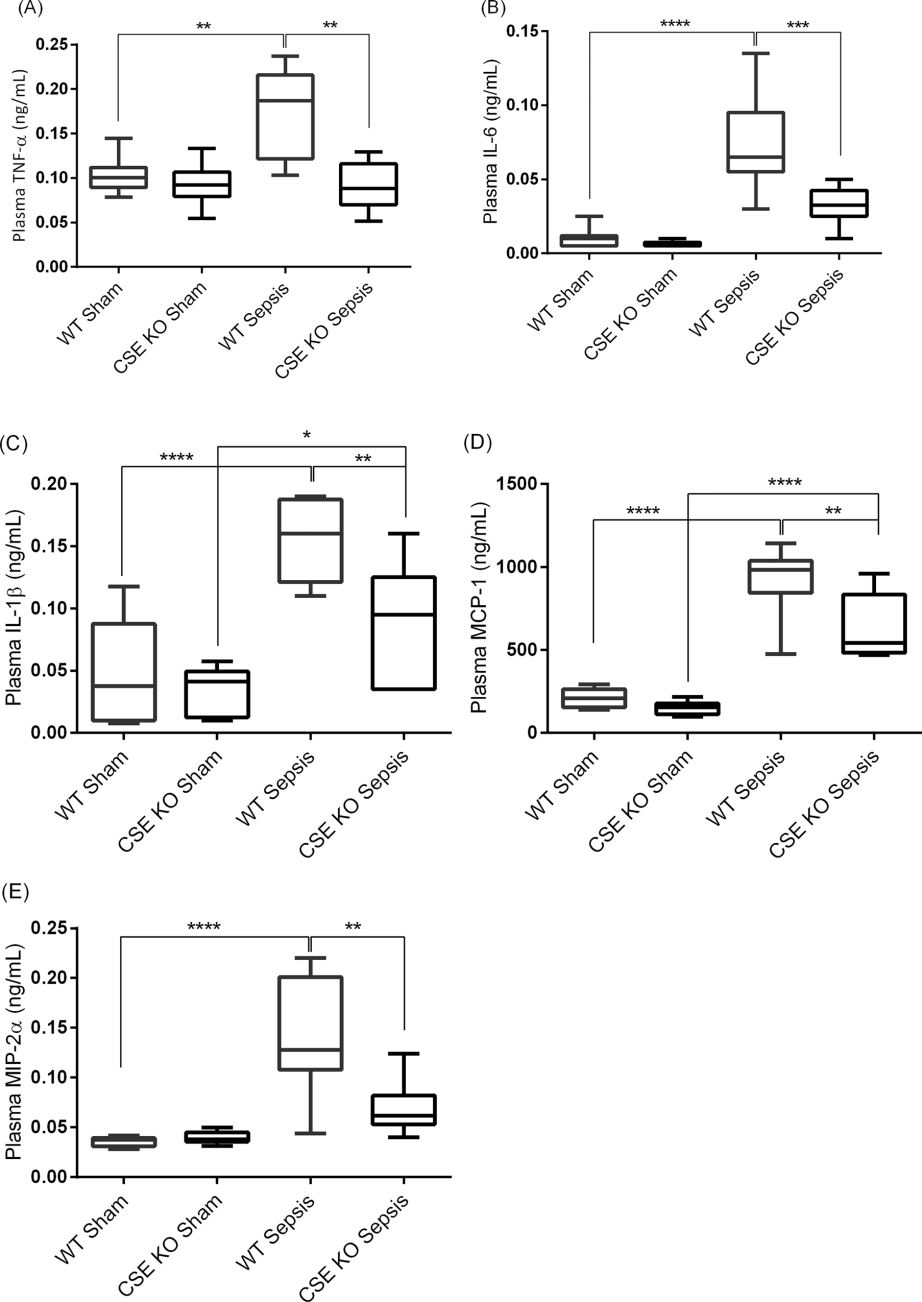
Effect of CSE Gene Deletion on Circulatory Inflammatory Mediators Following CLP Induced Sepsis. (A) Plasma TNF-α, (B) Plasma IL-6, (C) Plasma IL-1β, (D) Plasma MCP-1 and (E) Plasma MIP-2α. CLP induced sepsis significantly increased the plasma cytokines TNF-α (P<0.01), IL-6 (P<0.0001) and IL-1β (P<0.0001) and the chemokines MCP-1 (P<0.0001) and MIP-2α (P<0.0001) in WT mice compared to sham operation controls. Knockdown of the CSE gene protects mice against systemic inflammatory response by reducing pro-inflammatory TNF-α (P<0.01), IL-6 (P<0.05), IL-1β (P<0.001), MCP-1 (P<0.01) and MIP-2α (P<0.01) following CLP induced sepsis compared to WT sepsis mice. Results were expressed in ng/mL of plasma. Data represent the mean±standard deviation (n = 8). Data were analysed for Gaussian or Normal distribution using Shapiro-Wilk test. One-way ANOVA with post hoc Tukey’s test was performed to compare multiple groups. Statistical significance was assigned as *P<0.05; **P<0.01: and ***P<0.001.

WT CLP mice showed significantly higher levels of lung pro-inflammatory cytokines and chemokines compared with WT sham mice (Fig 7A–7E). CSE KO CLP mice displayed higher (or equivalent for TNF-α and MIP-2α) levels of those cytokines/chemokines in plasma compared with CSE KO sham mice, but the levels were much lower than those of WT CLP mice (Fig 7A–7E).

## Discussion

The role of H_2_S has been shown to play an important role in the pathogenesis of sepsis [3, 11]. PAG is the most commonly used inhibitor of H_2_S synthesis derived from CSE. Studies using PAG have suggested that CSE is a major contributor towards increased tissue and circulating H_2_S, as well as tissue H_2_S-synthesizing activity in several models of inflammation [3, 8, 11, 12, 37–39]. PAG inhibition of endogenous H_2_S synthesis has shown therapeutic effects in models of LPS-induced endotoxemia [7, 40], polymicrobial sepsis [12], and burn injury [8]. However, the use of PAG as a specific CSE inhibitor has been implicated as a possible confounding factor due to its nonspecific inhibition of other pyridoxal-5-phosphate-dependent enzymes. This emphasizes the importance of the present study; by using KO mice, we have specifically targeted the CSE-H_2_S pathway. In doing so, we have elucidated the direct effects of this pathway on polymicrobial sepsis without the possibility of the nonspecific effects as seen with PAG-based interventions.

In this study, we found that following sepsis there was an increase in the liver and lung CSE expression and H_2_S synthesizing activity which were correlated with augmented MPO activity as well as histological changes in the liver and lung. CSE deletion resulted in decreased synthesizing activity of H_2_S, decreased MPO activity in the liver and lung, and significant amelioration of the liver and lung injury.

In addition, we have shown that CSE deletion has reduced LSECs damage during sepsis. Activation of Kupffer cells during LPS-induced endotoxemia was associated with structural changes in LSECs [22, 41] but their role has yet to be demonstrated during sepsis. In addition, the role of CSE/H_2_S signalling pathway on LSECs fenestrae is not yet known. In this study, we have shown that increased LSECs damage is associated with CLP-induced polymicrobial sepsis as evidenced by LSECs defenestration (decreased diameter, frequency, and porosity of LSECs fenestration) and increase in the formation of the gaps in the LSECs. Gaps are large defects through the LSECs (usually more than 250 nm in diameter) that are associated with LSECs injury including oxidative stress and high pressure [33, 42, 43]. CSE gene deletion affords protection against LSECs injury by decreasing defenestration (increasing diameter, frequency and porosity of LSECs fenestration) and reducing gaps formation in the LSECs. Our results support previously published reports on LPS induced defenestration of LSECs during endotoxemia or *in vitro* exposure to the pseudomonal toxin, pyocyanin [22, 24, 44] even though we have used different experimental set up in this study compared to other previous studies, in particular we used a polymicrobial *in vivo* model of sepsis (a clinically relevant model of sepsis) rather than exposure of LSECs to a single bacterial toxin (lipopolysaccharide or pyocyanin). Also, previous studies make no mention of gaps, which is the novel finding in our study in terms of LSECs.

The mechanism by which CSE/H_2_S signalling pathway promotes inflammation and the inflammatory response in sepsis was also investigated. In sepsis, pathogens and their products trigger the inflammatory response by transcriptional activation of inflammatory genes, leading to the production of a large number of inflammatory mediators [16, 45]. Induction of multiple pro-inflammatory genes is mediated by the phosphorylation of ERK1/2 and subsequent activation of inducible transcriptional factor, such as NF-κB. Previously, it has been shown that H_2_S production during sepsis resulted in increased phosphorylation of ERK1/2 and degradation of IκB which allows nuclear translocation and induction of NF-κB [13]. Our study demonstrated that CSE/H_2_S signalling activation increased phosphorylation of ERK1/2 and subsequent translocation of p65 subunit of NF-κB into nucleus during sepsis. CSE gene deletion resulted in protection against liver, and lung injury and systemic inflammatory response by reducing phosphorylation of ERK1/2 and activation of NF-κB p65. In addition, CSE gene deletion not only attenuated sepsis associated activation of ERK1/2-NF-κB p65 signalling but also abolished the generation of cytokines TNF-α, IL-6, and IL-1β and chemokines MCP-1 and MIP-2α. These findings demonstrate that ERK1/2-NF-κB p65 signalling pathway may participate in CSE/H_2_S mediated inflammation during sepsis.

The use of CSE gene deletion approach to study CSE/H_2_S signalling role in sepsis associated liver, and lung injury and inflammation and systemic inflammatory response firmly establishes the importance of this signalling in inflammatory response of sepsis. In addition, for the first time, we have shown the CSE/H_2_S signalling mediated regulation of defenestration and gaps formation in the LSECs following sepsis. At the same time, some limitations of the current study need mention. First, this study is limited to only one time point (8 hours post CLP) and represents changes that occur at that time point. In earlier studies, however, PAG treatment, both prophylactic and therapeutic, has been shown to protect against inflammatory organ injury and prolong survival in sepsis [3]. Second, mechanisms of CSE/H_2_S mediated alterations of LSEC injury remain unclear. These will be the subject of future studies.

In conclusion, our study has demonstrated that deletion of the gene for CSE, an H_2_S synthesizing enzyme, decreased liver and lung injury and the associated systemic inflammatory response by decreasing the activation of ERK1/2-NF-κB p65 signalling and subsequent inflammation and an evidence of diminished tissue damage in the liver and lung during CLP-induced sepsis. CSE gene deletion also reduced sepsis induced injury to the LSECs as seen by decreased defenestration and reduced gaps formation in the LSECs.

## Supporting Information

S1 Fig CSE Protein Expression and H2S-Synthesizing Activity Following CLP Induced Sepsis. (XLSX)

S2 Fig Effect of CSE Gene Deletion on Liver and Lung MPO Activity and Organ Injury in Mice Following CLP Induced Sepsis. (XLSX)

S3 Fig Effect of CLP Induced Sepsis and CSE Gene Deletion on Liver Sieve Injury in Mice. (XLSX)

S4 Fig Effect of CSE Gene Deletion on the Phosphorylation of ERK1/2 and NF-kB p65 Activation in the Liver and Lung Following CLP Induced Sepsis. (XLSX)

S5 Fig Effect of CSE Gene Deletion on Liver Pro-inflammatory Cytokine and Chemokine Levels Following CLP Induced Sepsis. (XLSX)

S6 Fig Effect of CSE Gene Deletion on Lung Pro-inflammatory Cytokines and Chemokines. (XLSX)

S7 Fig Effect of CSE Gene Deletion on Circulatory Inflammatory Mediators Following CLP Induced Sepsis. (XLSX)
