# Correction: Cystathionine-Gamma-Lyase Gene Deletion Protects Mice against Inflammation and Liver Sieve Injury following Polymicrobial Sepsis

**DOI:** 10.1371/journal.pone.0183304

**Published:** 2017-08-10

**Authors:** Ravinder Reddy Gaddam, Robin Fraser, Alireza Badiei, Stephen Chambers, Victoria C. Cogger, David G. Le Couteur, Isao Ishii, Madhav Bhatia

The authors would like to correct Fig 4A. The incorrect images were used in Fig 4A. The images are also incorrectly labeled as “Liver CSE” and “GAPDH”. The lanes should be labeled “Liver p-ERK1/2” and “Liver ERK1/2” respectively. The authors have provided a corrected version of Fig 4 here.

**Fig 4:**
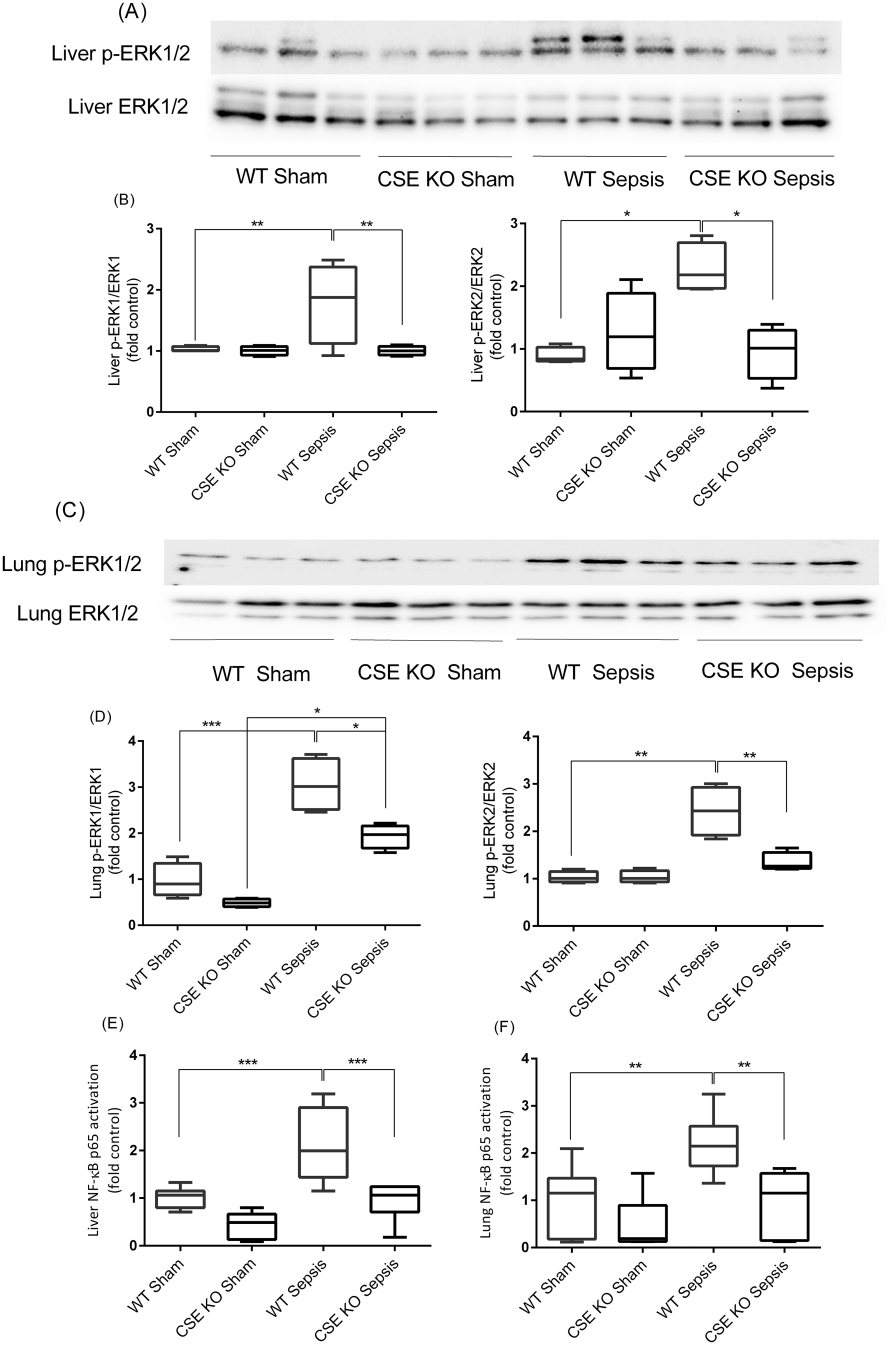
Effect of CSE Gene Deletion on the Phosphorylation of ERK1/2 and NF-kB p65 Activation in the Liver and Lung Following CLP Induced Sepsis. (A-B) Liver p-ERK1/2 expression. Phosphorylation of ERK1/2 (p-ERK1: P<0.01; p-ERK2: P<0.05) was increased following CLP induced sepsis in WT mice compared to sham operation controls. In CSE KO mice ERK1/2 phosphorylation (p-ERK1: P<0.01; p-ERK2: P<0.05) was reduced significantly following CLP induced sepsis compared to WT sepsis mice. (C-D) Lung p-ERK1/2 expression. Phosphorylation of ERK1/2 was increased (p-ERK1: P<0.001; p-ERK2: P<0.01) following CLP induced sepsis in WT mice compared to sham control. In CSE KO mice ERK1/2 phosphorylation was reduced significantly (p-ERK1: P<0.05; p-ERK2: P<0.01) following CLP induced sepsis compared to WT sepsis mice. Results were normalized with GAPDH and expressed as the relative fold increase of pERK1/2 expression compared with sham control. For western blot results, each lane represents a separate animal. The blots shown were representative of all animals in each group with similar results. (E-F) Liver and lung NF-κB p65 activation. Activation of NF-κB p65 (P<0.001) was increased following CLP induced sepsis in WT mice compared to sham control. In CSE KO mice NF-κB p65 activation (P<0.001) was decreased significantly following CLP induced sepsis compared to WT sepsis mice. Results were expressed as fold increase over control. Data represent the mean±standard deviation (n = 8). Data were analysed for Gaussian or Normal distribution using Shapiro-Wilk test. One-way ANOVA with post hoc Tukey’s test was performed to compare multiple groups. Statistical significance was assigned as *P<0.05; **P<0.01: and ***P<0.001.

The authors confirm that these changes do not alter their findings. The authors have provided raw, uncropped blots as Supporting Information.

## Supporting information

S1 File Uncropped Liver ERK1/2 blots. (TIF)

S2 File Uncropped Liver p-ERK1/2 blots. (TIF)
